# Identification of peptides sequence and conformation contributed to potential allergenicity of main allergens in yogurts

**DOI:** 10.3389/fnut.2022.1038466

**Published:** 2023-01-05

**Authors:** Meijia Huang, Fan Yang, Yong Wu, Xuanyi Meng, Linbo Shi, Hongbing Chen, Xin Li

**Affiliations:** ^1^State Key Laboratory of Food Science and Technology, Nanchang University, Nanchang, China; ^2^School of Food Science and Technology, Nanchang University, Nanchang, China; ^3^Sino-German Joint Research Institute (Jiangxi-OAI), Nanchang University, Nanchang, China; ^4^Jiangxi Province Key Laboratory of Food Allergy, Nanchang University, Nanchang, China; ^5^School of Basic Medical Sciences, Nanchang University, Nanchang, China

**Keywords:** yogurt, allergen, allergenicity, epitope, peptides

## Abstract

Yogurts provide a good source of nutrition and may induce tolerance in people with cow’s milk allergy (CMA). This study aimed to investigate the IgE-binding capacity of main allergens in the different yogurts which provide a reference for people with a high risk of CMA, and analyze the epitopes of major allergen peptides in yogurt. We assessed the degradation and the allergenic properties of major allergens in six commercial yogurts and fresh milk. The degradation of major allergens was analyzed by SDS-PAGE and RP-HPLC. Western blot and ELISA experiments detected allergenic characteristics by using specific sera. The results showed that β-lactoglobulin (Bos d 5) and α-lactalbumin (Bos d 4) were obviously degraded in yogurts but caseins were still present in abundance, which indicated that the proteases in yogurts were specific to whey proteins. IgE and IgG binding ability of major allergens were obviously reduced in yogurts, especially GuMi yogurt. In addition, 17 peptides of major allergens in GuMi yogurt were identified by LC-MS/MS and most of them were located in the interior of the spatial structure of proteins. Among them, 8 peptides had specific biological functions for health benefits, such as antibacterial, antioxidant, and ACE-inhibitory. We also found that 6 and 14 IgE epitopes of Bos d 5 and caseins were destroyed in GuMi yogurt, which could lead to the reduction of IgE-binding capacity. Meanwhile, peptides [Bos d 5 (AA15–40), Bos d 9 (AA120–151, AA125–151)] also preserved T cell epitopes, which might also induce the development of oral tolerance. Therefore, this study suggested that the sequence and conformation of peptides in yogurts contributed to hypoallergenicity.

## Graphical Abstract

Analysis of epitope peptides in yogurts. 
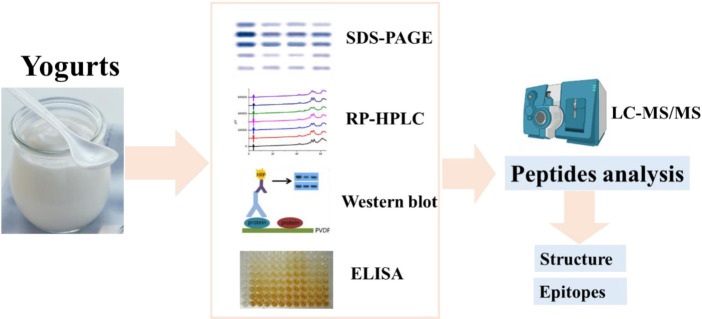

## 1. Introduction

Cow’s milk allergy (CMA) is the most common food allergy that seriously affects the quality of life (1). The incidence of CMA in some countries varies between 3.0 and 7.5% (2). The prevalence of CMA in infants has also reached 2.69% in China (3). The main allergens are caseins and whey proteins [β-lactoglobulin (Bos d 5, β-LG) and α-lactalbumin (Bos d 4, α-LA)] in cow’s milk (4). Caseins are divided into four subtypes, including α_S1_-casein (Bos d 9), α_S2_-casein (Bos d 10), β-casein (Bos d 11), and κ-casein (Bos d 12).

Yogurts have many health benefits and are an excellent source of proteins, vitamins and minerals (5). Previous clinical studies have shown that regular consumption of yogurt is associated with multiple health benefits, such as suppression of acute intestinal inflammation, and a reduced risk of high blood pressure and cardiovascular disease (6, 7). Yogurts are also a major source of live bacteria in the human diet, and these lactic acid bacteria (LAB) can produce proteinases during fermentation to hydrolyze proteins and affect the metabolism and the balance of endogenous flora. Tzvetkova et al. demonstrated that 21 LAB from the traditional yogurt could hydrolyze Bos d 4 and Bos d 5 to different degrees (8). Phromraksa et al. also reported that nine proteolytic bacteria obtained from fermented foods could degrade Bos d 5 and reduce the allergenicity of Bos d 5 (9). In addition to providing nutrition, yogurts are an excellent source of bioactive peptides. Bioactive peptides are released from proteins by proteolysis of bacteria in fermented foods (10). Peptides released in yogurts depend on proteolytic activity and fermentation conditions (11). Some evidence suggests that bioactive peptides in yogurts have functional activities, such as ACE-inhibition and antithrombotic activity (12–14). Meanwhile, yogurts have also played a role in food allergies, such as inducing tolerance and preventing allergies. Roduit et al. found that the introduction of yogurt in the first year of life had a protective effect on atopic dermatitis for allergic diseases (13). Shoda et al. showed that regular consumption of yogurts in infancy could prevent the development of food allergies based on the birth cohort study (15). Clinical studies have also found that yogurt could induce tolerance in most children with CMA (16, 17). Moreover, the consumption of yogurts is also gradually increasing in our lifespan.

The fermentation of dairy products not only reduces the antigenicity and allergenicity of milk proteins, but also makes the flavor good and produces many biologically active substances (18, 19). The proteases can produce peptides when hydrolyzing milk proteins, which have a great impact on the development and application of hypoallergenic dairy products (20). During the fermentation, the productive conditions and specific hydrolysis activities of yogurts might affect the structure and hydrolysis of proteins. The epitopes of major allergen peptides in yogurts have not been systematically investigated in previous studies. This study aimed to investigate the changes in the IgE-binding capacity of main allergens in the different yogurts and provide a reference for patients with a high risk of CMA. Meanwhile, the epitopes analysis of peptides in yogurt was carried out, which provided useful targets for peptide immune prevention and treatment in CMA.

## 2. Materials and methods

### 2.1. Reagents

Six yogurts and fresh milk were obtained from the supermarket. Among them, GuMi yogurt contained *Lactobacillus bulgaricus, Streptococcus thermophilus*, and *Lactococcus lactis* subsp. *Diacetyl*. JSD yogurt and HN yogurt contained *Lactobacillus bulgaricus, Streptococcus thermophilus, Streptococcus acidophilus*, and *Bifidobacterium lactis*. HR yogurt contained *Lactobacillus bulgaricus, Streptococcus thermophilus.* JA yogurt contained *Lactobacillus bulgaricus* and *Streptococcus thermophilus*. HS yogurt contained *Lactobacillus bulgaricus, Streptococcus thermophilus*, and *Lactobacillus plantarum*. The gelatin from cold water fish skin and 3,3,5,5-Tetramethylbenzidine (TMB) was purchased from Sigma Chemical Co. (St Louis, Mo, USA). The anti-α-LA/β-LG rabbit sera were obtained from our laboratory through immunized rabbits. The anti-caseins rabbit sera was obtained from Abbiotec Co. (San Diego, USA). A pool of 10 sera obtained from CMA patients with diverse symptoms was used in competitive enzyme-linked immunosorbent assay (ELISA) experiments and the information for allergic patients were listed in Table 1. Horseradish peroxidase (HRP)-labeled anti-rabbit IgG and biotinylated-labeled antihuman IgE were purchased from Sigma-Aldrich Chemical Co. (St Louis, USA). All other reagents were analytical grade.

**TABLE 1:** Information of patients with cow’s milk allergy.

Patient no.	Sex	Age (year)	Milk-related clinical symptoms	Milk S-IgE (kU_A_/L)
1	Female	10	AB	3.5
2	Female	13	P	1.0
3	Female	48	AR	3.2
4	Male	1	AS	2.9
5	Male	4	ND*	2.7
6	Male	4	U	5.9
7	Male	9	SU	56.8
8	Male	11	ND*	18
9	Male	12	L	43.7
10	Male	36	AR	3.7

AB, asthmatic bronchopneumonia; P, pneumonia; AR, allergic rhinitis; AS, asthma; ND*, not done; U, urticaria; SU, serum urticaria; L, leukocytosis.

### 2.2. Evaluation of major allergens in the yogurts

The commercial yogurts and fresh milk were analyzed by SDS-PAGE (12% polyacrylamide) and reversed phase high performance liquid chromatography (RP-HPLC). Then the samples were investigated on IgG and IgE binding abilities of proteins using competitive ELISA and Western blot (WB). Peptides in yogurts were identified by LC-MS/MS.

#### 2.2.1. SDS-PAGE

SDS-PAGE was performed on a MiniPROTEAN system (Bio-Rad Laboratories, USA) with 12% acrylamide separating gel and 4% acrylamide stacking gel. Protein samples (10 μg) were mixed with an equal volume of the loading buffer (100 mg SDS, 4 g sucrose, 2 mg bromophenol blue, 0.1 mL β-mercaptoethanol, and 2 mL of 50 mmol/L Tris-HCl in 10 mL ultrapure water) and then heated at 100°C for 5 min before loading. The time for running of gel was about 90 min with a constant current of 6–12 mA per gel. The gels were scanned using a SQ-GS800 scanning densitometer (Bio-Rad Laboratories, USA). After running the gels, gels were stained with Coomassie blue for 20 min and destained with a solution containing 5% methanol and 7.5% acetic acid.

#### 2.2.2. RP-HPLC

Reversed phase high performance liquid chromatography measured proteins in different yogurts, which a liquid chromatograph (Shimadzu, Japan) was used. The samples (20 μL, 1.5 mg/mL) were placed onto LC-20AT HPLC column (Symmetry C18, 5 μm particle size, 250 × 4.6 mm, Shimadzu) at a flow rate of 1.0 mL/min. Before the injection of samples, the column was equilibrated with 20% acetonitrile for 30 min. For solvent B which contained 0.1% (v/v) TFA in acetonitrile, multistep linear gradient elution was continuous from 5% at 0 min to 60% at 70 min. Ultraviolet detection was performed at 220 nm.

#### 2.2.3. WB analysis

The samples were resolved by 12% SDS-PAGE and subsequently electrotransfered to a nitrocellulose membrane. Blots were blocked with 3% gelatin (Sigma-Aldrich, St Louis, USA) blocking buffer (3 g gelatin in 100 mL TBS-T) for 2 h at room temperature and then incubated with specific sera (anti-caseins/α-LA/β-LG rabbit sera with a dilution of 1:5,000 for IgG and sera of allergic patients with 1:20 for IgE binding) overnight at 4°C. After washing three times with TBS-T (6.06 g Tris and 8.76 g NaCl in 1 L of ultrapure water, pH 7.5), the membranes were incubated with a secondary antibody (HRP-labeled anti-human IgG/biotinylated-labeled anti-human IgE) for 1 h at room temperature. For the IgE binding experiments, avidin was added to react with biotin and incubated for 1 h at 37°C before the chromogenic substrate. Afterward, the immune reaction was visualized by the enhanced chemiluminescence detection system (Bio-Rad Co., Hercules, USA).

#### 2.2.4. Competitive ELISA

The IgE binding ability of yogurts was identified by competitive ELISA (21). Briefly, 1 μg/mL of skim milk in 0.05 M carbonate buffer (pH 9.6) was used to coat a 96-well microtiter plate (100 μL/well) at 4°C overnight. The plates were washed three times. Each well was blocked for 1 h at 37°C with 3% gelatin in 0.02 M PBS (0.2 g KH_2_PO_4_, 2.9 g Na_2_HPO_4_⋅12H_2_O, 8.0 g NaCl, and 0.2 g KCl in 1,000 mL distilled water, pH 7.4). 60 μL of sera (allergic patients’ pooled sera with a dilution of 1:150 for IgE) and 60 μL of samples with different inhibitor concentrations (1, 10, 50, 100, and 250 μg/mL) were mixed and incubated for 1 h at 37°C. Subsequently, 100 μL of the preincubated mixture was added to each well and incubated for 1 h at 37°C. The plate was washed, and 100 μL of the diluted 1:5,000 antisera (biotinylated-labeled anti-human IgE) with PBS (0.02 M) was added to the wells and incubated at 37°C for 1 h. Then avidin was added as an additional step before the chromogenic substrate. After washing again, 100 μL of the substrate solution (TMB) was added to each well. The chromogenic reaction was terminated by adding 50 μL of 2 M sulfuric acid per well. Absorbance was determined at 450 nm.

#### 2.2.5. LC-MS/MS

The samples were separated using the HPLC liquid system Easy nLC at nanoliter flow rates. Buffer A is 0.1% formic acid in ultrapure water, and B is 0.1% formic acid in acetonitrile (84% in acetonitrile). The chromatographic column was equilibrated with 95% of liquid A, and the sample was loaded from the autosampler to the loading column (Thermo Fisher Scientific Acclaim PepMap100, 100 μm × 2 cm, C18), and then passed through the analytical column (Thermo Scientific EASY column, 10 cm, ID75 μm, 3 μm). Then samples were chromatographically separated and analyzed by mass spectrometry using a Q-Exactive mass spectrometer. The detection method was positive ion, and the primary mass spectrometry resolution was 70,000 at 200 m/z. The dynamic exclusion time was 60 S. The secondary MS resolution was 17,500 at 200 m/z. The final data was queried in MaxQuant software, and the amino acid composition of the final peptides was confirmed by comparison.

### 2.3. The prediction of allergenicity

The peptides in yogurts were submitted to the tool AllerTop 2.0,^1^ which is a server for the allergenicity prediction based on the physicochemical properties of amino acid sequences (22, 23).

### 2.4. Mapping of epitopes on allergens

The 3D structure of the Bos d 5 was obtained from the Protein Data Bank.^2^ The spatial structure of Bos d 9, Bos d 10, Bos d 11, and Bos d 12 were predicted by the online software I-TASSER.^3^ Then the peptides of allergens were labeled in different colors with the PyMOL visualization software (The PyMOL Molecular Graphics System, Version 4.6).

### 2.5. Statistical analysis

*T*-test was used for statistical analysis, and then GraphPad Prism 5.0 (GraphPad Software Inc., San Diego, CA, USA) was used for Student’s *t*-test. Different letters represent significant differences (*p* < 0.05).

## 3. Results and discussion

### 3.1. Degradation of main allergens in yogurts

**FIGURE 1:**
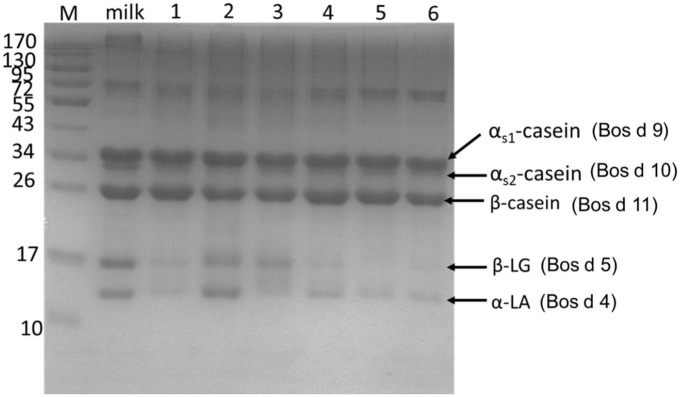
SDS-PAGE showed the proteins profile of yogurts. Lane 1: GuMi yogurt; Lane 2: JSD yogurt; Lane 3: HR yogurt; Lane 4: HN yogurt; Lane 5: JA yogurt; Lane 6: HS yogurt. Lane M: prestained marker.

**FIGURE 2:**
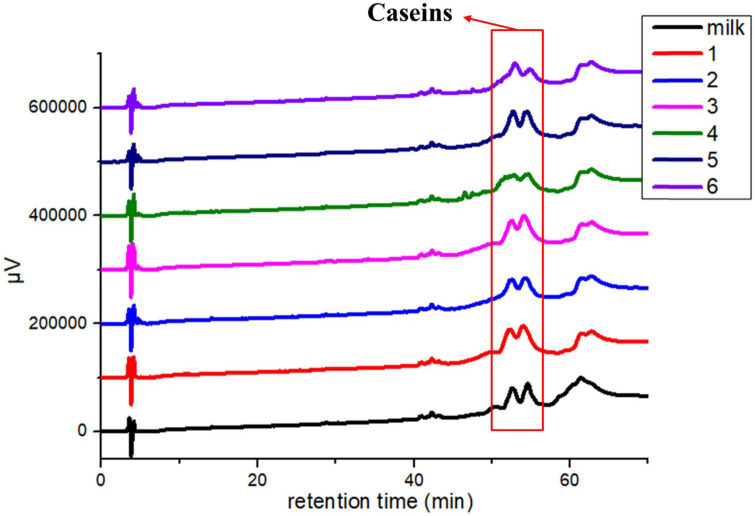
Reverse phase-HPLC profiles of yogurts. Lane 1: GuMi yogurt; Lane 2: JSD yogurt; Lane 3: HR yogurt; Lane 4: HN yogurt; Lane 5: JA yogurt; Lane 6: HS yogurt.

In the study, the degradation of major allergens in yogurts was detected by SDS-PAGE (Figure 1) and RP-HPLC (Figure 2). In Figure 1, the results showed that the degradation of major allergens in yogurts was different according to protein bands. In particular, the degradation degree of Bos d 4 and Bos d 5 was obviously different in these yogurts. In addition to JSD yogurt (lane 2), Bos d 4 and Bos d 5 were obviously degraded in yogurts compared with fresh milk according to the change of protein bands in Figure 1. And there was no obviously change in the protein bands of caseins, which suggested that caseins in yogurts was hardly degraded. These results indicated that proteases in yogurts contributed to hydrolyze the whey proteins, but not caseins fraction. It can be speculated that Bos d 4 and Bos d 5 were broken down into different peptides or free amino acids, while caseins were most stable in these yogurts. Similar results were found in previous studies, in which caseins also were present in abundance in yogurt (14). In addition, caseins are also a heat-stable protein (24). RP-HPLC further confirmed this result based on the peak of caseins, which were stable. It indicated that the hydrolysis of proteins was limited in the fermentation process compared with digestion *in vitro* (14). Nguyen et al. have proved similar results as well, in which the degradation of major proteins in yogurts was limited through SDS-PAGE analysis (25). It is generally known that milk proteins are hydrolyzed by LAB during yogurt fermentation. Previous studies have also shown that the main proteins of dairy products could be hydrolyzed by the strains from yogurts (8, 11). The fermentation conditions and bacteria in yogurts could all affect the degree of hydrolysis of milk proteins.

### 3.2. Analysis of allergic characteristics of main allergens in yogurts

**FIGURE 3:**
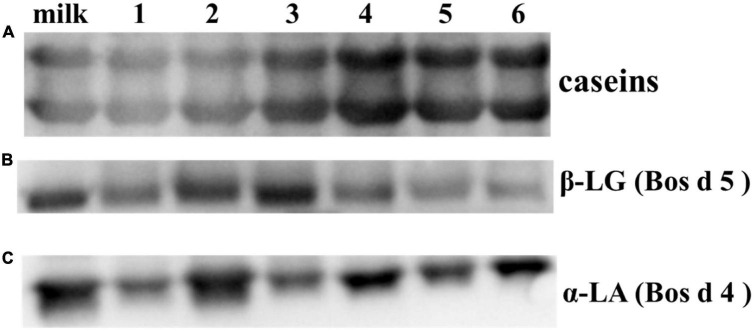
Western blot analysis detected IgG binding ability of main allergens **(A)** caseins; **(B)** Bos d 5; **(C)** Bos d 4 in yogurts by using anti-caseins/Bos d 5/Bos d 4 specific sera of rabbit.

**FIGURE 4:**
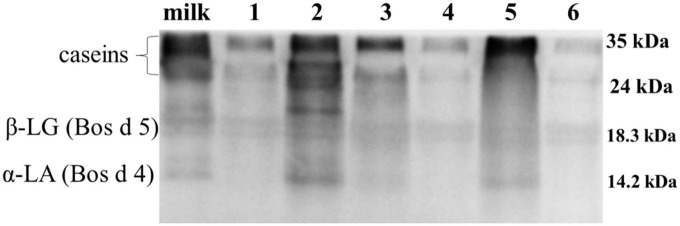
Western blot analysis detected IgE binding ability of main allergens in yogurts by using sera of allergic patients. Lane 1: GuMi yogurt; Lane 2: JSD yogurt; Lane 3: HR yogurt; Lane 4: HN yogurt; Lane 5: JA yogurt; Lane 6: HS yogurt.

The antigenicity of proteins is caused by specific sequences of allergen epitopes that can bind to specific antibodies (26). In our study, WB was mainly used to detect linear epitopes in samples, which the sample preparation of yogurts has been completely or partially denatured. Stable linear epitopes can provide an upfront basis for subsequent peptide vaccine production and preparation of monoclonal antibodies. Therefore, the initial identification of linear epitopes was very necessary. The IgG binding ability of yogurts was measured using rabbit sera for antigenicity evaluation by WB as shown in Figure 3. In Figure 3A, the IgG binding ability of casein in HR yogurt (Lane 3), HN yogurt (Lane 4), JA yogurt (Lane 5), and HS yogurt (Lane 6) was increased according to protein bands compared to fresh milk. The increased antigenicity (IgG binding ability) might be due to exposure of IgG binding epitopes in casein. In Figure 3B, the IgG binding capacity of Bos d 5 was reduced in GuMi yogurt (Lane 1), HN yogurt (Lane 4), JA yogurt (Lane 5), and HS yogurt (Lane 6), which indicated that some IgG epitopes were destroyed. In Figure 3C, the IgG binding ability of Bos d 4 in GuMi yogurt (Lane 1), HR yogurt (Lane 3), and JA yogurt (Lane 5) was reduced, which might destroy or bury the IgG epitopes of Bos d 4. Obviously, fermented yogurt disrupted the structure and epitope of protein to varying degrees, which was consistent with previous results (27). Overall, the IgG binding ability of GuMi yogurt was the lowest. These results indicated that fermented yogurts disrupted allergen epitopes. The increased antigenicity of allergens in yogurts resulted from the exposure of allergen IgG epitopes, which were buried internally in unfermented milk. Studies have indicated that the fermentation of LAB could reduce the antigenicity of proteins (27, 28). Pessi et al. also indicated that *L. rhamnosus* GG could degrade casein and release peptides, thus affecting the epitopes (29). The potential allergenicity of allergens was assessed by detecting the IgE binding capacity of proteins with the sera of allergic patients (30). Disruption of protein structure and hidden epitopes could affect the potential allergenicity of proteins (24). WB analysis detected IgE binding ability of main allergens in yogurts by using sera of allergic patients about CMA (Figure 4). Compared with fresh milk, the IgE binding capacity of GuMi yogurt (Lane 1), HR yogurt (Lane 3), HN yogurt (Lane 4), JA yogurt (Lane 5), and HS yogurt (Lane 6) was reduced according to the protein bands in Figure 4. It was also evident that there were the stable, highly conserved linear epitopes in yogurts. These results also provide preliminary basic research for the design of epitope vaccine and preparation of monoclonal antibody with highly conserved peptide epitopes (31). Similarly, Anna et al. found that linear epitopes were important for allergen identification through SDS-PAGE and WB under denaturing conditions (32). Wang et al. also found that two highly conserved linear epitopes of Hexon Protein could help design new structure-based epitope vaccines or therapeutic vaccines (33). The results indicated that the IgE epitopes of major allergens were destroyed or buried during the fermentation process of yogurts. The IgE binding capacity of JSD yogurt was increased, which might be due to the exposure of hidden epitopes. It indicated that the proteases by fermentation could hydrolyze and disrupt epitopes to varying degrees in different yogurts.

**FIGURE 5:**
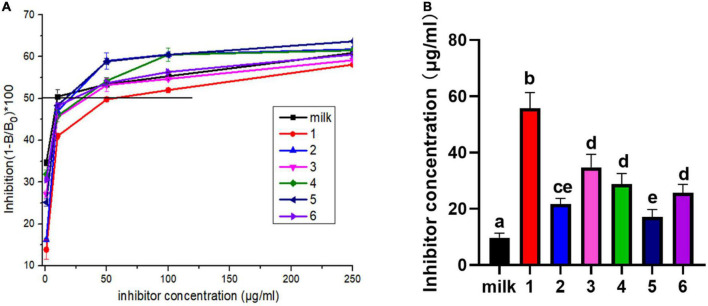
IgE binding ability of yogurts was defined by competitive ELISA **(A)**. The inhibitor concentration corresponds to IC50 values of yogurts **(B)**. Lane 1: GuMi yogurt; Lane 2: JSD yogurt; Lane 3: HR yogurt; Lane 4: HN yogurt; Lane 5: JA yogurt; Lane 6: HS yogurt (Different letters represent significant differences, *p* < 0.05).

The competitive ELISA assay was also used to detect the IgE binding ability of protein in Figure 5. IC_50_ is the competitive concentration of allergen when antibody binding has been inhibited by 50%, which a high IC_50_ value indicates low allergenicity (21). In the Figure 5A, the calculated IC_50_ values of the samples from fresh milk, JA yogurt, JSD yogurt, HS yogurt, HN yogurt, HR yogurt, and GuMi yogurt were gradually increased. Compared with fresh milk, the statistical analysis indicated that the IC_50_ of JA yogurt, JSD yogurt, HS yogurt, HN yogurt, HR yogurt, and GuMi yogurt were significantly increased in the Figure 5B (*p* < 0.05). Among them, the IC_50_ of GuMi yogurt was the highest, indicating that the IgE binding ability was the lowest. The IC_50_ of HR yogurt, HN yogurt, and HS yogurt was significantly lower than GuMi yogurt (*p* < 0.05). The IC_50_ of JSD yogurt and HS yogurt was the lowest, indicating that their IgE binding ability was the highest among these yogurts. These results demonstrated that the IgE binding ability after the fermentation was reduced compared with fresh milk. Based on the above results, GuMi yogurt was found to have the lowest antigenicity and IgE binding ability. Minjing Yao et al. indicated that fermentation by *L. rhamnosus* GG could significantly reduce the antigenicity and human residual IgE-binding capacity of Bos d 4, Bos d 5, α-casein, and β-casein in the reconstituted milk (34). Kordesedehi et al. also found that *Enterococcus faecium* could hydrolyze Bos d 9 in many sites including the main allergen epitopes, in which IgE binding capacity was significantly reduced (35). During the fermentation process, the release of proteases and peptidases results in the effective hydrolysis of milk proteins with a possibility for cleavage of epitopes. Nevertheless, it is also possible that the peptides are further cleaved into smaller peptides by peptidase, and hidden epitopes may be exposed.

### 3.3. Identification of peptides released by GuMi yogurt and epitopes analysis

Based on the above results, GuMi yogurt with the lowest antigenicity and IgE binding ability was selected for the identification of the peptides by LC-MS/MS. 17 peptides from GuMi yogurt were detected by LC-MS/MS in Table 2. Of note, there were no peptides obtained from Bos d 4. It might be the amount of peptides was too low to detection. The detected peptides derived from Bos d 5, Bos d 9, Bos d 10, Bos d 11, and Bos d 12 were 3, 8, 2, 2, and 2, respectively. Among them, Bos d 9 AA 23–34 and α_s1_-casein AA80–90 had antihypertensive and antithrombotic activity, respectively (36, 37). Bos d 5 AA 41–46 and Bos d 12 AA 25–34 had the hypocholesterolemic function and opioid, respectively. Bos d 5 AA15–40 and Bos d 10 AA 174–181 also had the activities of antioxidant, ACE-inhibitory, and Bos d 10 AA 198–205 and Bos d 12 AA 69–86 had antimicrobial activity (37, 38). Eight bioactive peptides with specific functions were produced in the yogurt, which was beneficial to health.

**TABLE 2:** Peptides identified by LC-MS/MS in the yogurt.

Protein sequence (protein fragment)	Predicted allergenicity of peptides	Allergenic sequence*	References
VAGTWYSLAMAASDISLLDAQSAPLR (Bos d 5 AA15–40)	Yes	H (1–16, 31–60) N (21–40)	(46–49)
VYVEELKPTPEGDLEILLQK (Bos d 5 AA41–60)	No	N (41–60), H (56–70, 58–77)	
TPEVDDEALEKFDK (Bos d 5 AA124–137)	Yes	H (121–140, 127–152)	
HQGLPQEVLNENLLR (Bos d 9 AA8–22)	Yes	H (6–20, 11–35, 16–35)	(46, 47, 50)
HIQKEDVPSER (Bos d 9 AA80–90)	Yes	H (76–90)	
EDVPSER (Bos d 9 AA84–90)	No	H (76–90)	
FFVAPFPEVFGK (Bos d 9 AA23–34)	Yes	H (11–35, 16–35)	
FFVAPFPEVFGKEK (Bos d 9 AA23–36)	No	H (11–35, 16–35)	
LHSMKEGIHAQQK (Bos d 9 AA120–132)	No	H (126–140)	
LHSMKEGIHAQQKEPMIGVNQELAYFYPELFR (Bos d 9 AA120–151)	Yes	H (139–154), N (126–140)	
EGIHAQQKEPMIGVNQELAYFYPELFR (Bos d 9 AA125–151)	Yes	H (139–154), N (126–140)	
FALPQYLK (Bos d 10 AA 174–181)	Yes	H (165–188)	(51)
TKVIPYVR (Bos d 10 AA 198–205)	Yes	H (191–200)	
AVPYPQRDMPIQAFLL (Bos d 11 AA 177–192)	Yes	H (167–178, 173–184)	(52)
DMPIQAFLLYQEPVLGPVRGPFPIIV (Bos d 11 AA 184–209)	Yes	N (185–208)	
YIPIQYVLSR (Bos d 12 AA 25–34)	No	H (9–26, 21–44, 16–35)	(47, 52)
SPAQILQWQVLSNTVPAK (Bos d 12 AA 69–86)	No	H (67–78)	

Predicted allergenicity of peptides were predicted by the tool AllerTop 2.0 (https://www.ddg-pharmfac.net/AllerTOP/). Allergy sequences are the IgE epitopes that have been previously reported in the literature.

*H indicates that the allergenic epitope has been hydrolyzed and *N* not cleaved.

Table 2 also showed the characteristics of detected peptides, including predicted allergenicity and position of the allergenic sequence. The results (Table 2) showed that 20 IgE epitopes of allergens are disrupted through previously reported literature. Among them, 6, 6, 2, 2, 4 allergic epitopes were also disrupted in Bos d 5, Bos d 9, Bos d 10, Bos d 11, and Bos d 12, respectively. Meanwhile, these peptides were also submitted to the bioinformatics tool of AllerTop 2.0 for allergenicity prediction, and the results found that 11 peptides had the potential allergenicity while 6 peptides did not (Table 2). These might be factors that lead to changes in the allergenicity of yogurts. Of note, Bos d 5 (AA15–40) and Bos d 9 (AA 120–151, AA 125–151) retained T cell epitopes which were reported in previous studies (39, 40). It was reported that peptides preserved T cell epitopes by several studies, which could suppress allergic responses and were also critical for the induction of oral tolerance (41–43). Previous studies have also indicated that peptides containing T cell epitopes could induce oral tolerance of CMA (44, 45). These all suggested that T cell epitopes might provide a practical approach for the prevention and treatment of allergy and tolerance induction. In our study, fermented yogurts not only destroyed allergen epitopes, but also generated tolerogenic peptides that might induce the development of tolerance. But further *in vivo* studies are needed to determine the ability of these peptides to induce tolerance.

**FIGURE 6:**
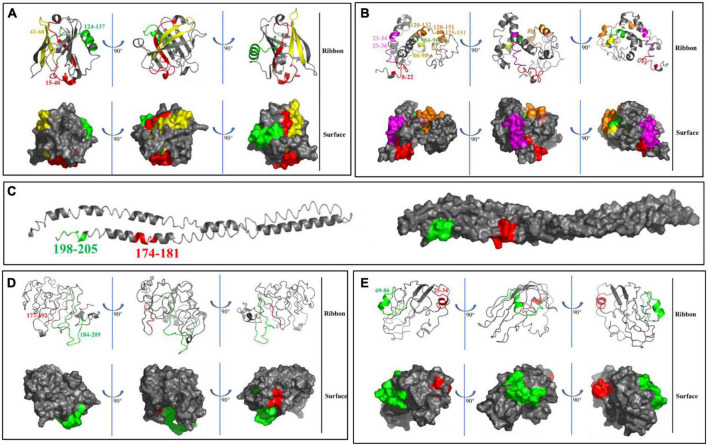
Mapping of peptides on the spatial structure of Bos d 5 **(A)**, Bos d 9 **(B)**, Bos d 10 **(C)**, Bos d 11 **(D)**, and Bos d 12 **(E)** by using ribbon and surface diagrams based on Pymol visualization software.

Interestingly, lysine (K) and arginine (R) residues were at the carboxyl terminus for 15 peptides. Therefore, we speculated that proteases with specific cleavage sites might be produced in yogurt. Meanwhile, the conformational structure of 17 peptides obtained in GM yogurt were displayed in Figure 6. Among them, 3 peptides were mapped on the spatial structure of Bos d 5 (Figure 6A), and 8, 2, 2, 2 peptides were mapped on Bos d 9, Bos d 10, Bos d 11, and Bos d 12 to the spatial structure predicted by I-TASSER, respectively (Figures 6B–E). It was found that most peptides were located in the interior of the protein, so these peptides were not easily hydrolyzed and retained. Moreover, 14 peptides are composed of α helices and random coils. It is generally known that the structure of proteins or peptides is closely related to the allergenicity. The analysis of the structure and epitopes of peptides could allow us to conduct targeted studies in the future.

## 4. Conclusion

In our study, we compared the differences in allergenicity of major allergens in six commercial yogurts firstly and found that GuMi yogurt had the lowest antigenicity and allergenicity. We found that 17 peptides of major allergens in GuMi yogurt were identified and most of them were located in the interior of the spatial structure of proteins, which speculated these peptides might be encapsulated and not easily cleaved. Among them, 6 peptides did not have the potential allergenicity by prediction. Meanwhile, 6 and 14 IgE epitopes of Bos d 5 and caseins were also hydrolyzed in GuMi yogurt, which could reduce allergic reactions. Of note, the peptides [Bos d 5 (AA15–40), Bos d 9 (AA120–151, AA125–151)] also preserved T cell epitopes, which might also induce the development of oral tolerance. All of these factors contribute to the hypoallergenicity of yogurt. Therefore, GuMi yogurt with low allergenicity provides a reference for patients with a high risk of CMA and analysis of the allergenic properties of peptides in yogurts is helpful to the development of oral peptide vaccines. However, further *in vivo* trials are needed to confirm. Interestingly, no peptide in Bos d 4 was detected, which deserves further exploration.

## Data availability statement

The original contributions presented in this study are included in the article/supplementary material, further inquiries can be directed to the corresponding author.

## Author contributions

MH was responsible for designing the experiments and writing the manuscript. FY and YW helped to analyze the data. XM conducted some experiments. LS and HC helped to conduct the experimental design. XL conducted the experimental design and revised the manuscript. All authors contributed to the article and approved the submitted version.

## Footnotes

1
https://www.ddg-pharmfac.net/AllerTOP/

2
https://www.rcsb.org/structure/1BEB

3
https://zhanggroup.org//I-TASSER/
